# Thermal Effects and Small Signal Modulation of 1.3-μm InAs/GaAs Self-Assembled Quantum-Dot Lasers

**DOI:** 10.1007/s11671-010-9798-4

**Published:** 2010-09-26

**Authors:** HX Zhao, SF Yoon, CZ Tong, CY Liu, R Wang, Q Cao

**Affiliations:** 1 School of Electrical and Electronic Engineering, Nanyang Technological University, Singapore 639798, Republic of Singapore; 2 Photonics Group, Edward S. Rogers Sr. Department of Electrical and Computer Engineering, University of Toronto, 27 King's College Circle, Toronto, ON Canada; 3 Institute of Solid State Physics, Technical University of Berlin, Hardenbergstr. 36, 10623 Berlin, Germany

**Keywords:** Molecular beam epitaxy, Temperature, Modulation, Quantum-dots, Semiconductor lasers

## Abstract

We investigate the influence of thermal effects on the high-speed performance of 1.3-μm InAs/GaAs quantum-dot lasers in a wide temperature range (5–50°C). Ridge waveguide devices with 1.1 mm cavity length exhibit small signal modulation bandwidths of 7.51 GHz at 5°C and 3.98 GHz at 50°C. Temperature-dependent *K*-factor, differential gain, and gain compression factor are studied. While the intrinsic damping-limited modulation bandwidth is as high as 23 GHz, the actual modulation bandwidth is limited by carrier thermalization under continuous wave operation. Saturation of the resonance frequency was found to be the result of thermal reduction in the differential gain, which may originate from carrier thermalization.

## Introduction

High-temperature stability in laser operation is an essential characteristic required for the long-wavelength semiconductor lasers in optical communication systems. Realization of uncooled high-speed operation of 1.3-μm quantum-dot (QD) lasers has attracted intensive research interests due to its application in optical communication. Over the past decade, promising dynamic properties of QDs such as large differential gain, high cut-off frequency, and small chirp were reported in devices with emission wavelength less than 1.2 μm [1]. Improved temperature characteristics of QD lasers, such as temperature-invariant threshold current [1], high characteristic temperature (*T*_o_) [2], and linewidth enhancement factor [3], have been realized through *p*-doping technique. However, quantum-dots emitting at 1.3 μm and above have not fulfilled the initial expectation of improved temperature-insensitive modulation bandwidths, which have largely remain below 12 GHz [4]. With the increase in QD size and the strain effect of the cap layer, the self-assembled (SA) InAs/GaAs QDs can emit at 1.3 μm. The energy levels are still discrete. However, the number of energy level increases and the level separation, especially for holes, becomes much narrower (8–11 meV for hole) than that in the short-wavelength QDs. This results in significant hole thermalization [5]. Other problems reported in the 1.3-μm SA QDs include the finite GaAs barrier and thin wetting layer [6]. These disadvantages consequently lead to the temperature-sensitive performance observed in 1.3-μm QD lasers, such as the low characteristic temperature at or above room temperature [7] and strong temperature-dependent maximum gain. Fiore et al. [8] have studied the effects of intradot relaxation on the *K*-factor and differential gain of quantum-dot lasers. Deppe et al. [9] have reported the role of density of states, especially thermalization of holes due to their closely spaced discrete energy levels. This limits the modulation speed of QDs with deep confinement potentials such as the 1.3-μm InAs/GaAs QDs. Many theoretical [8,9] and experimental [3,10,11] investigations have been performed to study the bandwidth limitations in long-wavelength QD lasers. According to these investigations, *K*-factor [8,11] has been recognized to be one of the limiting factors for the modulation bandwidth of QD lasers, which accounts for the effect of photon lifetime, differential gain, and nonlinear gain compression factor. Despite the theoretical and experimental investigations on the effect of differential gain on the DC performance of 1.3-μm QD laser and directly modulated uncooled 1.3-μm QD laser [12], the effect of carrier thermalization on the high-speed performance of 1.3-μm QD laser has not been analyzed systematically. Obviously, the modulation speed (or bandwidth) of the 1.3-μm QD lasers should be temperature-dependent due to the temperature-sensitive gain profile of QDs. As there are few investigations on the effect of temperature on the bandwidth of 1.3-μm QD lasers, a study on this aspect will provide greater understanding on the differential gain and carrier dynamics in long-wavelength QD lasers.

In this paper, we investigate the influence of thermal effects on the high-speed modulation characteristics of 1.3-μm InAs/GaAs QDs by studying the temperature-dependent small signal modulation behavior. The effects of temperature on the *K*-factor, differential gain, and nonlinear gain compression will be presented here.

## Experimental Details

**Figure 1:**
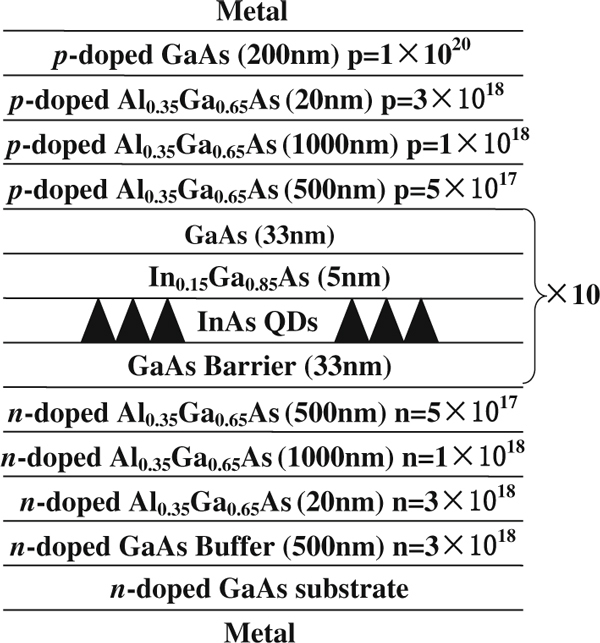
**The schematic diagram of the InAs/GaAs ten-layer QD laser structure**.

The ten-layer self-assembled InAs/GaAs QD laser structure, as shown in Figure 1, was grown on GaAs (100) substrate by molecular beam epitaxy (MBE). The structure consists of QD active region sandwiched between two 1.5-μm C- and Si-doped Al_0.35_Ga_0.65_As cladding layers. The active layer comprises 2.3 monolayer (ML) of InAs QDs capped by a 5-nm In_0.15_Ga_0.85_As layer. A 33-nm GaAs layer is used to separate the two QD layers [13]. The wafer was processed into 4-μm-wide ridge waveguide (RWG) lasers by standard photolithography process and wet chemical etching at room temperature (RT) [14]. Ridge height of approximately 1.3 μm was obtained before the pulsed anodic oxidation (PAO) process. A 200 ± 5 nm-thick oxide layer was formed by PAO method, whose experimental setup can be found in [15]. Subsequently, p-contact layers (Ti/Au, 50/300 nm) were deposited by electron beam evaporation, while n-contact layers (Ni/Ge/Au/Ni/Au, 5/20/100/25/300 nm) were deposited on the backside of the substrate following lapping down to ~100 μm. Finally, the wafer was cleaved into laser bars and the cleaved facets were left uncoated. The devices were mounted p-side down on a heat sink for measuring the small signal modulation characteristics. The small signal modulation response of the QD lasers was measured under continuous wave (CW) biasing condition using a vector network analyzer (VNA), a high-speed photoreceiver and laser diode current source. A thermoelectric temperature controller was used to regulate and monitor the device temperature during measurements.

## Results and Discussions

**Figure 2:**
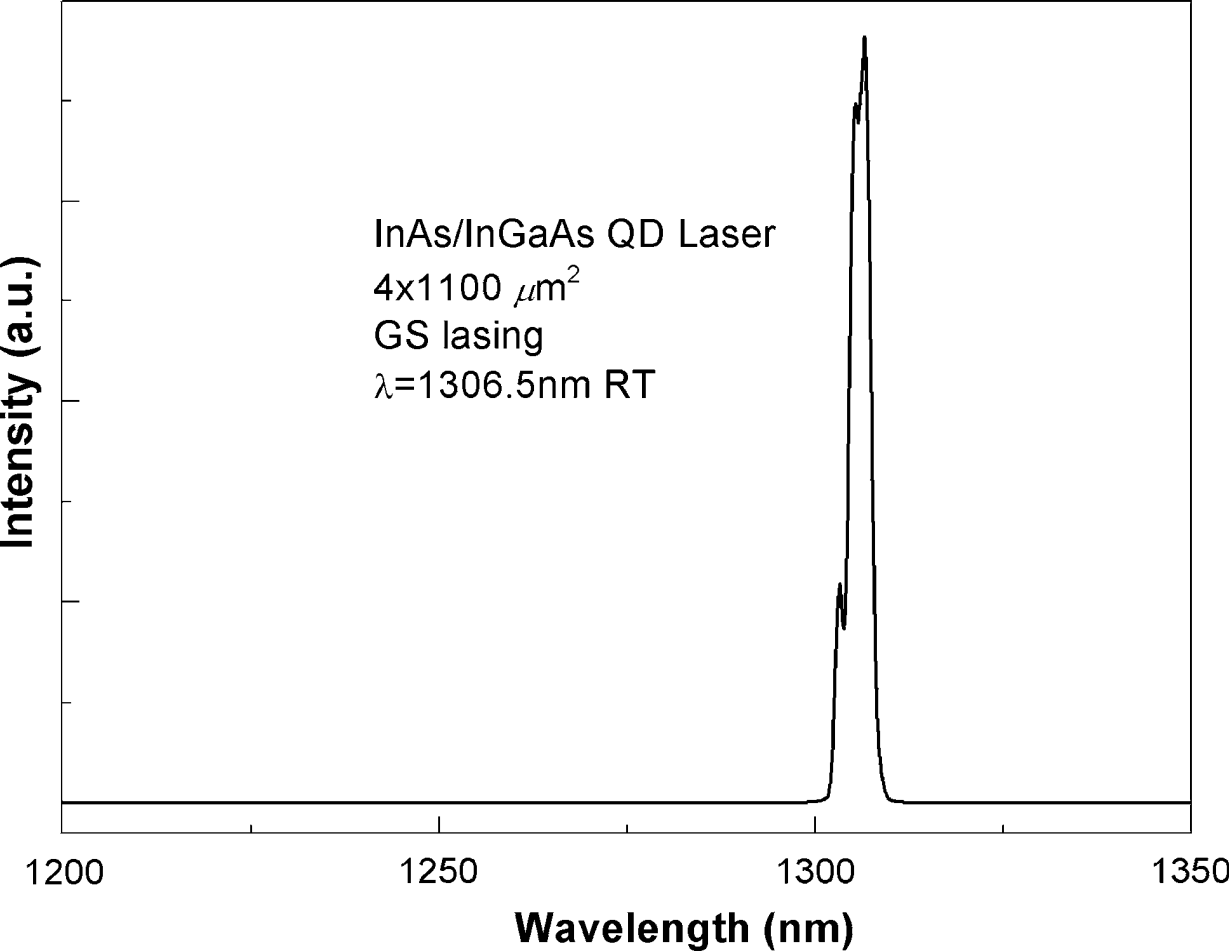
**The lasing spectrum from the InAs/InGaAs QD laser (4 × 1,100 μm^2^) with injection current of 100 mA at RT**.

**Figure 3:**
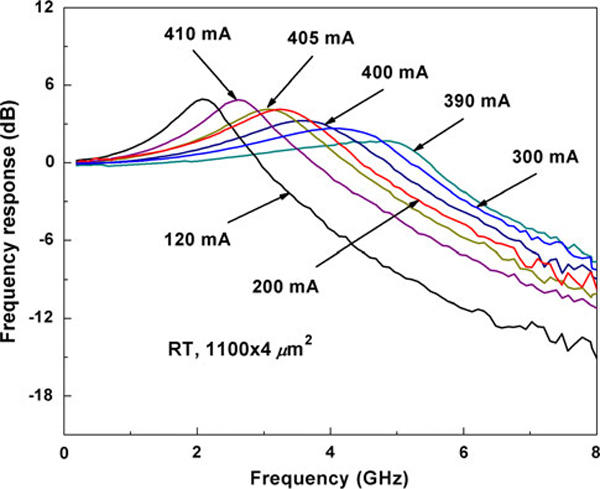
**Small signal modulation response measured at RT under different injection current levels**.

The measured CW Power-Current performance of a device with cavity length of 1.1 mm shows that the threshold current (*I*_th_) and slope efficiency are 55 mA and 0.27 W/A at room temperature, respectively. Maximum output power of 96 mW occurred at injection current of 395 mA. Figure 2 shows the lasing spectrum of the laser device under injection current of 100 mA at RT for verification. The lasing wavelength is centered at 1,306.5 nm. Furthermore, no lasing at excited state was observed. Characteristic temperature *T*_o_ is around 41 K from 5 to 50°C. The small signal modulation response under different injection current levels is shown in Figure 3. At room temperature, the highest bandwidth of 6.1 GHz was obtained at injection current level of 390 mA. For injection current more than 395 mA, the resonance frequency *f*_r_ decreases with increasing injection current. This is because, there are two competing factors affecting the resonance frequency: (1) increase in resonance frequency with injection current and (2) decrease in resonance frequency due to internal heating. Therefore, when injection current increases higher than 395 mA, the internal heating resulted from the increased current becomes dominant and leads to the decrease in resonance frequency. The small signal modulation response was further fitted into a transfer function that accounts for the intrinsic response of the laser as well as the extrinsic effects [16]:

(1) $$H(f)=\text{const}\cdot \frac{f_{\text{r}}^{2}}{f_{\text{r}}^{2}-f^{2}+j\frac{f}{2\pi }\gamma }♉\frac{1}{1+j\frac{f}{f_{\text{p}}}}$$

where *f*_r_ is the resonance frequency, *γ* is the damping rate, and *f*_p_ is the parasitic cut-off frequency. From the fitting, we obtained values of the damping rate *γ* and resonance frequency *f*_r_ at different bias currents. The parasitic cut-off frequency is almost temperature-independent and only restricts the bandwidth minimally. According to the plot of *f*_r_ vs. the square root of the normalized bias current (*I* – *I*_th_)^1/2^, the slope (known as *D*-factor or modulation efficiency) is obtained to be 0.28 GHz/mA^1/2^ at RT. The relationship between resonance frequency and damping rate defines the *K*-factor, which is 0.83 ns at RT. Furthermore, the *K*-factor is directly related to the damping-limited bandwidth (*f*_3dB, damping_) by [16]:

(2) $$f_{3\text{dB,damping}}=\frac{2\sqrt{2}\pi }{K}$$

The internal quantum efficiency (*η*_*i*_) and internal optical loss (*α*_*i*_) of the devices were estimated to be 51% and 4 cm^-1^ by measuring lasers with different cavity length (1–3 mm) [17,18]. The internal quantum efficiency and internal optical loss exhibit weak dependency on temperature. With the values of internal quantum efficiency and internal optical loss, the differential gain (d*g/*d*n*) and nonlinear gain compression factor (*ε*) are extracted. The gain derivatives with respect to the carrier population defines differential gain, while the nonlinear gain compression factor is used to describe the gain dependence on the photon density. From the value of the *D*-factor, the differential gain is obtained to be 11.1 × 10^-15^ cm^2^ at RT, which is almost ten times higher than that reported in literature [19] (differential gain of 1 × 10^-15^ cm^2^ at 300 K for a device emitting at 1,263 nm). The nonlinear gain compression factor is determined to be 12 × 10^-16^ cm^3^ at RT. Note that the results are different from that reported recently [20]. We believe that the differences are due to the different device dimensions considered since the performance depends strongly on the device dimensions [21,22].

**Figure 4:**
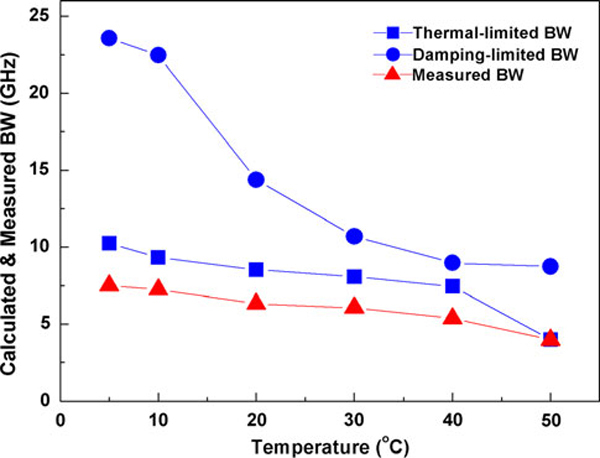
**Calculated thermal- (*squares*) and damping-limited (*circles*) bandwidth (*BW*) and plot of the measured (*triangles*) bandwidth at different temperatures**.

**Figure 5:**
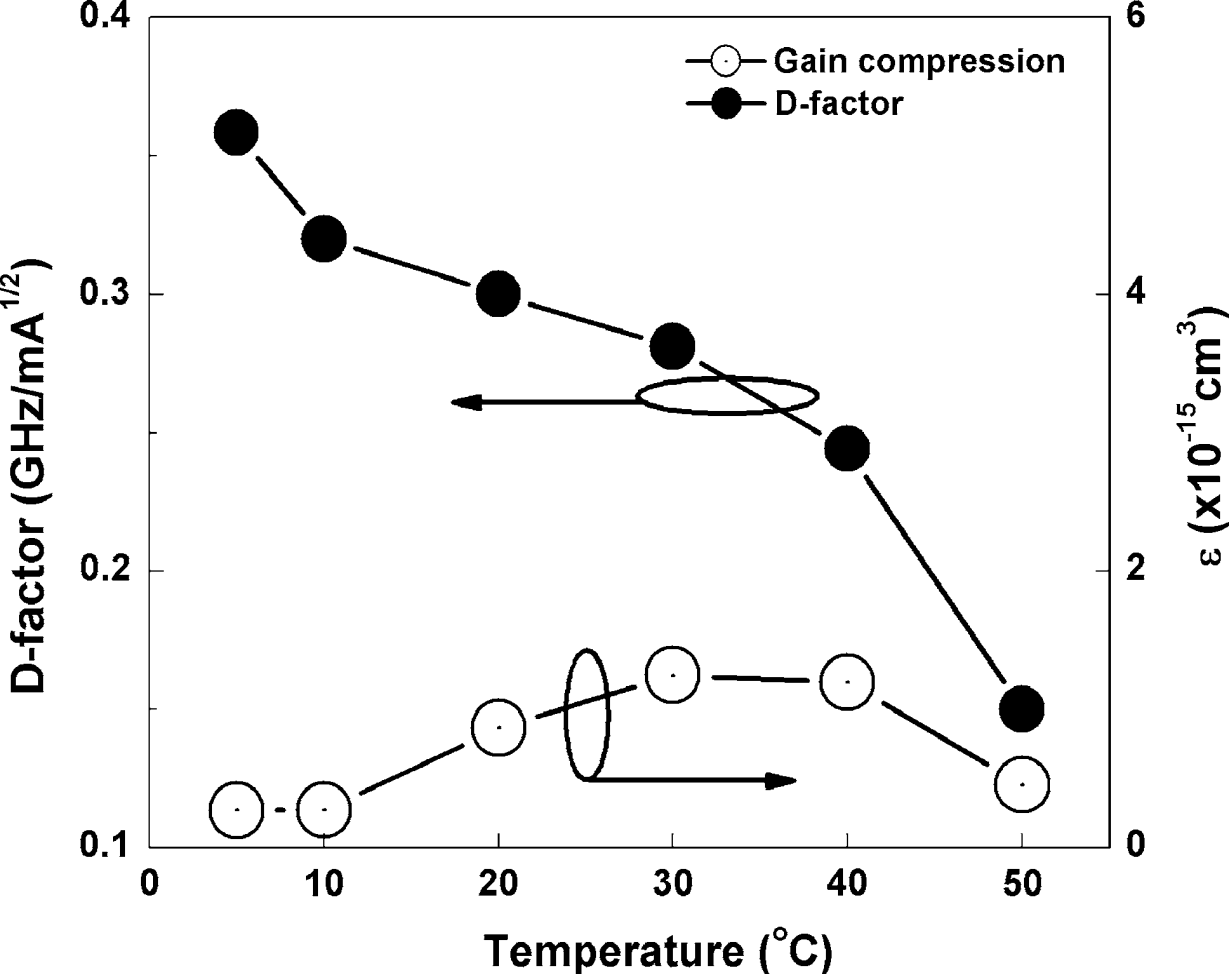
**The dependence of *D*-factor (*solid circle*) and nonlinear gain compression (*hollow circle*) on temperature**.

**Figure 6:**
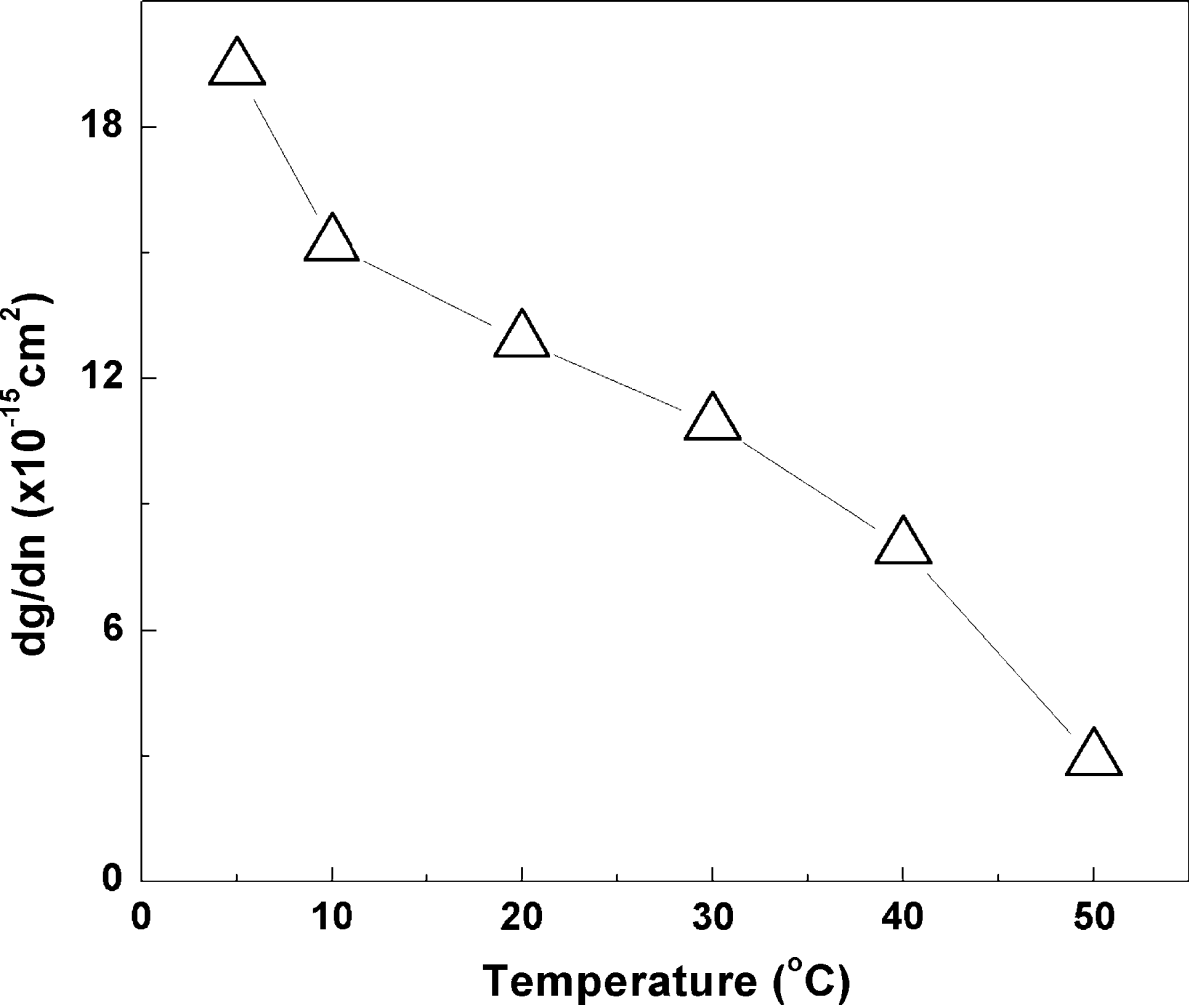
**The differential gain at different temperatures**.

**Figure 7:**
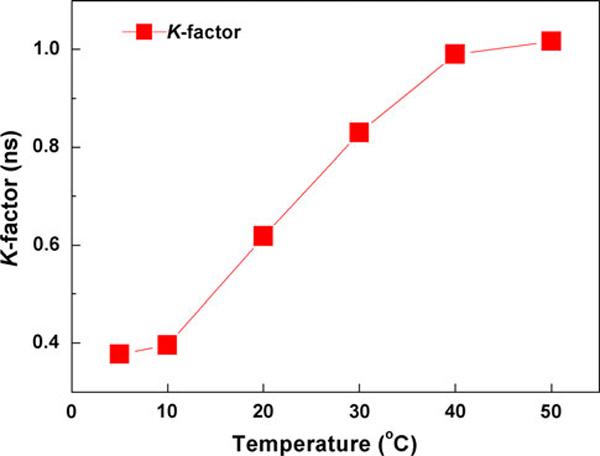
**Plot of temperature-dependence of *K*-factor**.

Measurements of direct small signal modulation of the QD laser were carried out from 5 to 50°C. Figure 4 shows the maximum measured (triangles) bandwidth (*f*_3dB, measured_) as function of temperature. The maximum measured bandwidth decreases almost linearly with temperature as temperature increases from 5 to 50°C. The highest *f*_3dB, measured_ of 7.51 GHz occurred at 5°C. The *D*-factor is 0.36 GHz/mA^1/2^ at 5°C and 0.15 GHz/mA^1/2^ at 50°C as shown in Figure 5 (solid circles). The differential gain from 5 to 50°C decreases following increase in temperature as shown in Figure 6. Figure 7 shows the calculated *K*-factor of the QD laser as function of temperature. There is a significant increase in the *K*-factor as temperature increases. The calculated *K*-factor increases approximately by a factor of three over the temperature range of 5–50°C.

From (2), the *f*_3dB,damping_ of the QD laser is 23 GHz at 5°C and 8.9 GHz at 50°C, which is limited by the carrier-capture time and modal gain via the *K*-factor [11,23]. This shows that the 1.3-μm InAs/GaAs QD lasers can potentially operate at very high frequencies. However, our experimental data shows much lower bandwidth. This can be attributed to the thermal effects, i.e., the thermal saturation of photon number (*S*_o_) at roll-over current injection due to the self-heating. It could also be caused by serious hole thermalization due to the closely spaced hole levels in 1.3-μm InAs QDs. Considering the dependence of bandwidth on resonance frequency, this suggests that the saturation of the bandwidth is caused by saturation of the photon density. Saturation of the photon density could possibly be caused by the strong gain compression. Meanwhile, the nonlinear gain compression factor is in the order of 10^-16^ cm^3^ and shows a relatively weak dependence on temperature [refer to Figure 5 (hollow circle)]. The *ε* · *S*_o_ product is less than 0.1, which suggests that the effect of the gain compression on the resonance frequency is relatively small. At relatively small damping effects, the thermal-limited bandwidth (*f*_3dB,thermal_) is related to *f*_r_ by [16]:

(3) $$f_{3\text{dB,thermal}}=\sqrt{1+\sqrt{2}}f_{\text{r},\max}$$

where *f*_r, max_ is the maximum resonance frequency at a constant temperature. The *f*_r, max_ of 6.6 GHz at 5°C and 2.5 GHz at 50°C would give a thermal-limited bandwidth of 10 GHz and 3.9 GHz (squares in Figure 4), respectively. This suggests that the main limitation on the bandwidth might be due to the decrease in differential gain, which may result from the thermal effects related to carrier thermalization in the multi-stack quantum-dots. The origin of the temperature-dependent differential gain is currently under investigation. The incorporation of *p*-type modulation doping and tunnel injection might be useful to improve the QD laser performance by reducing the thermal effects.

Finally, the calculated intrinsic damping-limited bandwidth (squares) and thermal-limited bandwidth (circles) are shown in Figure 4 in comparison with the experimental results *f*_3dB, measured_ (triangles). The thermal-limited *f*_3dB,thermal_ is in close agreement with the experimental results, indicating that the bandwidth measured in this study was limited by thermal effects.

## Conclusion

In conclusion, we have studied the influence of thermal effects on the small signal modulation characteristics of undoped InAs/GaAs QD lasers. The role of temperature-dependent differential gain and nonlinear gain compression factor in determining the frequency bandwidth was investigated. Calculation of the temperature-dependent bandwidth of the undoped QD laser shows close agreement between the thermal-limited bandwidth and the measurement results. The bandwidth of the undoped InAs/GaAs QD lasers is mainly limited by thermal effects, which may result from carrier thermalization in the undoped QD laser structure.

## References

[B1] Fathpour S Mi Z Bhattacharya P IEEE Photonics Technol Lett 2005 17 2250 10.1109/LPT.2005.857242

[B2] Shchekin OB Deppe DG IEEE Photonics Technol Lett 2002 14 1231 10.1109/LPT.2002.801597

[B3] Cong DY Martinez A Merghem K Ramdane A Provost JG Fischer M Krestnikov I Kovsh A Appl Phys Lett 2008 92 191109 10.1063/1.2929384

[B4] Kim SM Wang Y Keever M Harris JS IEEE Photonics Technol Lett 2004 16 377 10.1109/LPT.2003.823088

[B5] Shchekin OB Deppe DG Appl Phys Lett 2002 80 2758 10.1063/1.1469212

[B6] Xu DW Yoon SF Tong CZ IEEE J Quantum Electron 2008 44 879 10.1109/JQE.2008.925136

[B7] Mukai K Nakata Y Otsubo K Sugawara M Yokoyama N Ishikawa H IEEE J Quantum Electron 2000 36 472 10.1109/3.831025

[B8] Fiore A Markus A IEEE J Quantum Electron 2007 43 287 10.1109/JQE.2006.890399

[B9] Deppe DG Huang H Shchekin OB IEEE J Quantum Electron 2002 38 1587 10.1109/JQE.2002.805246

[B10] Gyoungwon P Shchekin OB Deppe DG IEEE J Quantum Electron 2000 36 1065 10.1109/3.863959

[B11] Ishida M Hatori N Akiyama T Otsubo K Nakata Y Ebe H Sugawara M Arakawa Y Appl Phys Lett 2004 85 4145 10.1063/1.1811789

[B12] Todaro MT Salhi A Fortunato L Cingolani R Passaseo A De Vittorio M Della Casa P Ghiglieno F Bianco L IEEE Photonics Technol Lett 2007 19 191 10.1109/LPT.2006.890045

[B13] Cao Q Yoon SF Liu CY Ngo CY Nanoscale Res Lett 2007 2 303 10.1007/s11671-007-9066-4

[B14] Wang R Tong CZ Yoon SF Liu CY Zhao HX Cao Q IEEE Electron Device Lett 2009 30 1311 10.1109/LED.2009.2033718

[B15] Yuan S Jagadish C Kim Y Chang Y Tan HH Cohen RM Petravic M Dao LV Gal M Chan MCY Li EH O JS Zory PS Jr IEEE J Sel Top Quantum Electron 1998 4 629 10.1109/2944.720473

[B16] Coldren LA Corzine SW 1995 Wiley

[B17] Kirstaedter N Schmidt OG Ledentsov NN Bimberg D Ustinov VM Egorov AY Zhukov AE Maximov MV Kopev PS Alferov ZI Appl Phys Lett 1996 69 1226 10.1063/1.117419

[B18] Kim J Chuang SL IEEE J Quantum Electron 2006 42 942 10.1109/JQE.2006.880380

[B19] Kuntz M Ledentsov NN Bimberg D Kovsh AR Ustinov VM Zhukov AE Shernyakov YM Appl Phys Lett 2002 81 3846 10.1063/1.1521572

[B20] Zhao HX Yoon SF Ngo CY Wang R Tong CZ Liu CY Cao Q IEEE Photonics J 2010 2 630 10.1109/JPHOT.2010.2052843

[B21] Liu CY Qu Y Yuan S Yoon SF Appl Phys Lett 2004 85 4594 10.1063/1.1824180

[B22] Liu CY Yoon SF Fan WJ Uddin A Yuan S IEEE Photonics Technol Lett 2006 18 791 10.1109/LPT.2006.871697

[B23] Klotzkin D Bhattacharya P IEEE J Lightwave Technol 1999 17 1634 10.1109/50.788569

